# Capture and selective release of multiple types of circulating tumor cells using smart DNAzyme probes†

**DOI:** 10.1039/c9sc04309h

**Published:** 2020-01-09

**Authors:** Qianying Zhang, Wenjing Wang, Shan Huang, Sha Yu, Tingting Tan, Jian-Rong Zhang, Jun-Jie Zhu

**Affiliations:** State Key Laboratory of Analytical Chemistry for Life Science, School of Chemistry and Chemical Engineering, Nanjing University Nanjing 210023 China jrzhang@nju.edu.cn jjzhu@nju.edu.cn; School of Chemistry and Life Science, Nanjing University Jinling College Nanjing 210089 China; State Key Laboratory of Agricultural Microbiology, College of Science, Huazhong Agricultural University Wuhan 430070 China; Department of Laboratory Medicine, Nanjing Drum Tower Hospital, The Affiliated Hospital of Nanjing University Medical School Nanjing 210008 China

## Abstract

The effective capture, release and reanalysis of circulating tumor cells (CTCs) are of great significance to acquire tumor information and promote the progress of tumor therapy. Particularly, the selective release of multiple types of CTCs is critical to further study; however, it is still a great challenge. To meet this challenge, we designed a smart DNAzyme probe-based platform. By combining multiple targeting aptamers and multiple metal ion responsive DNAzymes, efficient capture and selective release of multiple types CTCs were realized. Sgc8c aptamer integrated Cu^2+^-dependent DNAzyme and TD05 aptamer integrated Mg^2+^-dependent DNAzyme can capture CCRF-CEM cells and Ramos cells respectively on the substrate. With the addition of Cu^2+^ or Mg^2+^, CCRF-CEM cells or Ramos cells will be released from the substrate with specific selectivity. Furthermore, our platform has been successfully demonstrated in the whole blood sample. Therefore, our capture/release platform will benefit research on the molecular analysis of CTCs after release and has great potential for cancer diagnosis and individualized treatment.

## Abstract 2

Smart DNAzyme probes realized the capture and selective release of multiple circulating tumor cells.

## Graphical abstract

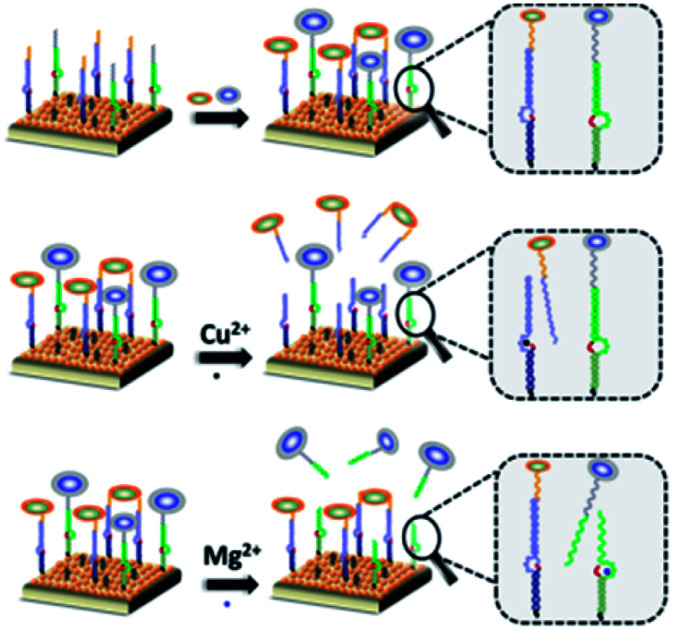

## Introduction

Circulating tumor cells (CTCs) are a class of cancer cells present in peripheral blood which cast off from the primary tumor into peripheral blood.^1,2^ The detection and isolation of CTCs are of vital importance for the early diagnosis of cancer, monitoring of therapeutic efficacy and evaluation of treatment.^3–6^ Since the amount of CTCs is extremely exiguous in patients' blood,^7–9^ there is an imperative demand to capture and enrich CTCs with high efficiency accordingly. In the last few years, multitudinous systems have been reported for the separation and enrichment of CTCs such as magnetic isolation technology,^10–13^ microfluidic technology^14–17^ and flow cytometry.^18,19^ Most of the above methods relied on a single CTC marker to capture CTC. Considering that the expression of biomarkers on the surface of CTCs would change after CTCs undergo epithelial–mesenchymal transition, a single marker-based methodology is deficient to capture all CTCs in real blood samples, hindering further clinical studies.^20–23^ To meet this challenge, platforms that are able to capture and identify multiple types of CTCs based on multiple surface markers are highly desired and under rapid development.^24,25^

Aptamers are a class of functional nucleic acid molecules, which can bind a range of targets specifically, such as proteins and intact cancer cells, after forming a unique tertiary structure.^26,27^ Aptamers are more friendly to prepare and easy to modify due to the simple chemical structure and excellent stability.^28,29^ In view of these advantages, aptamers have been extensively applied to biosensing and drug-delivery therapeutics.^30–35^ Besides, aptamers have also been used for CTC capture and exhibit excellent capture efficiency.^24,25,36^

In addition to CTC capture, the following release step is also critical, because it enables further molecular characterization studies of CTCs to be performed to obtain important information of the primary tumor, which promotes the progress of individualized therapies of tumors.^14,37^ Methods employed to release CTCs include nuclease release,^24,38,39^ photothermal release,^40–42^ electrochemical release,^43,44^ enzymatic release,^45^ magnetic release^46^ or mechanosensitive release;^47^ however, selective release of multiple CTCs has not been reported. Considering that the cultivation and reanalysis of released CTCs are significant, it is necessary to realize the selective release of multiple CTCs captured.

DNAzymes are functional nucleic acid molecules with catalytic properties produced by *in vitro* selection.^48–53^ Based on the specific properties of recognizing metal ions and undergoing catalytic reactions of nucleic acid cleavage, DNAzymes have been widely used as sensor elements for metal ion sensing and as a cyclic signal amplification tool for biomarker identification both *in vitro* and *in vivo*.^54–61^ Furthermore, DNAzymes have been utilized as gate keepers to realize the encapsulation and release of molecules.^62–64^ Inspired by these studies, we speculate that DNAzymes can be used for selective release of multiple types of CTCs assisted by metal ions.

**Fig. 1:**
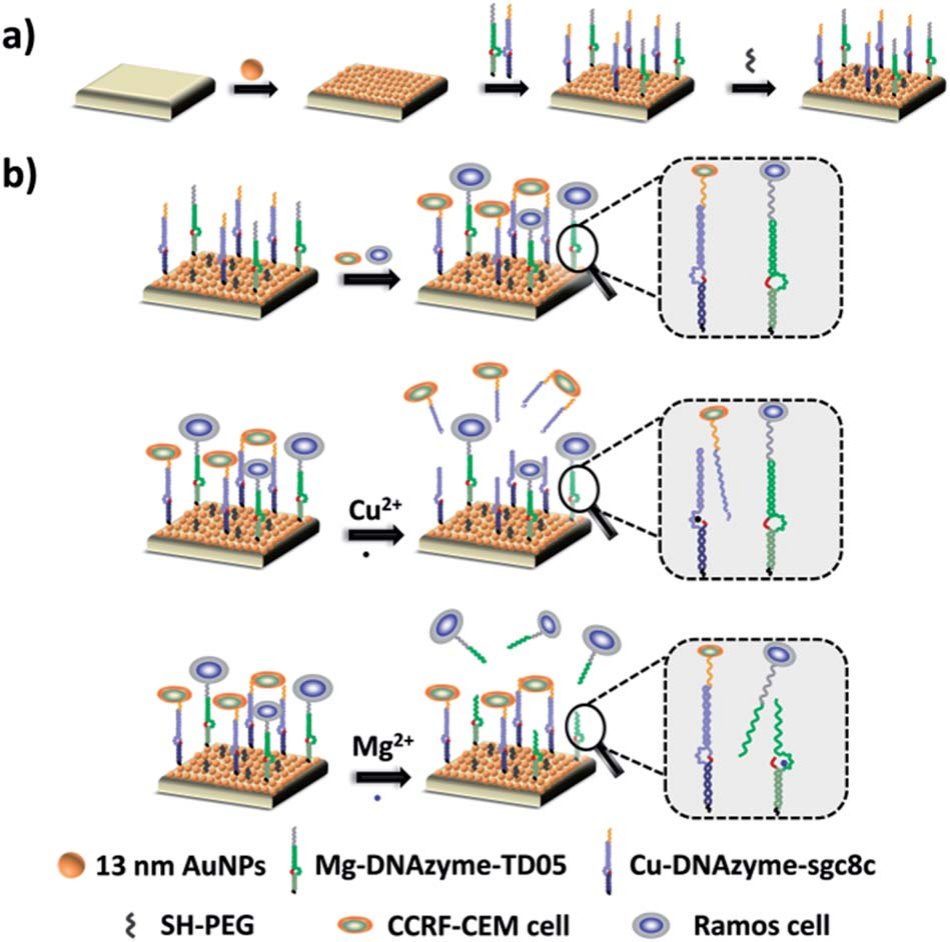
Schematic of the proposed strategy based on the combination of DNAzymes and aptamers for simultaneous capture and selective release of multiple CTCs. (a) Fabrication of the capture/release platform; (b) illustration of the capture of CTCs by aptamers and the selective release of CTCs by metal ions.

Herein, we established a platform to capture and selectively release multiple CTCs efficiently based on smart DNAzyme probes, as illustrated in Fig. 1. In our study, we used CCRF-CEM cells and Ramos cells as the model cells. The Sgc8c aptamer was chosen to capture CCRF-CEM cells, T-cell acute lymphoblastic leukemia (ALL) cell line, while the TD05 aptamer was used to bind Ramos cells which refer to a human Burkitt's lymphoma cell line.^65,66^ The capture element for CCRF-CEM cells (Cu^2+^-DNAzyme-sgc8c) was composed of the Cu^2+^-dependent DNAzyme strands and the substrate strands integrated with sgc8c aptamers. Similarly, the capture element for Ramos cells (Mg^2+^-DNAzyme-TD05) was composed of the Mg^2+^-dependent DNAzyme strands and the substrate strands integrated with TD05 aptamers. CCRF-CEM cells and Ramos cells can be captured by sgc8c aptamers and TD05 aptamers, respectively. The addition of Cu^2+^ as the cofactor catalyzed the cleavage of the substrate strands of Cu^2+^-dependent DNAzyme, releasing CCRF-CEM cells. Similarly, the addition of Mg^2+^ as the cofactor catalyzed the cleavage of substrate strands of Mg^2+^-dependent DNAzyme, releasing Ramos cells. Consequently, this platform realizes the capture of multiple CTCs by multiple aptamers, as well as the selective release of CTCs with the addition of Cu^2+^ or Mg^2+^.

## Results and discussion

### Principle of the capture/release platform

The principle of our strategy to capture and selectively release multiple CTCs is shown in Fig. 1. The substrate was first modified with AuNPs, which provided more attachment sites for the capture element than the flat gold substrate. Cu^2+^-DNAzyme-sgc8c and Mg^2+^-DNAzyme-TD05 were modified to capture CCRF-CEM cells and Ramos cells respectively. As shown in Fig. 1b, after CCRF-CEM cells and Ramos cells were captured, Cu^2+^ was used to trigger the Cu^2+^-dependent DNAzyme strands to catalyze the cleavage of the substrate strands to release CCRF-CEM cells, while Ramos cells cannot be released. Correspondingly, when Mg^2+^ was used to trigger the Mg^2+^-dependent DNAzyme strands to catalyze the cleavage of the substrate strands, only Ramos cells were released from the substrate. In the samples containing both CTCs and even in blood samples, our platform can capture multiple CTCs efficiently. The two captured CTCs can be released from the substrate selectively after the addition of Cu^2+^ or Mg^2+^. Compared with the methods using anti-EPCAM antibody as a single marker^11,14,40,47^ (Table S1†), our method can simultaneously capture multiple CTCs in blood samples, avoiding the incomplete capture due to change of CTCs' surface biomarkers after epithelial–mesenchymal transition. In addition, compared with the method to capture multiple CTCs using multiple aptamers as markers^24,25^ (Table S1†), selective release of multiple CTCs was achieved which makes multiple CTC typing possible and more valuable downstream molecular information of CTCs can be obtained. Our method not only captures CTCs in the blood more efficiently, but selectively releases multiple CTCs to facilitate further downstream molecular analysis of CTCs.

### Fabrication and characterization of the capture platform

**Fig. 2:**
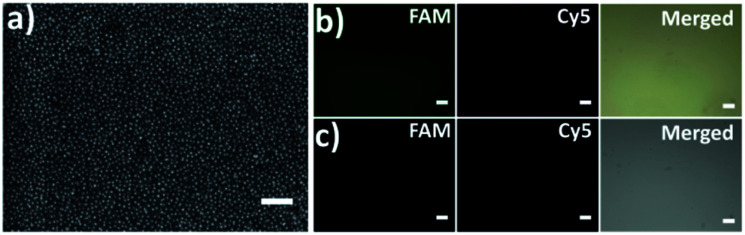
(a) Scanning electron microscopy (SEM) image of the 13 nm AuNP modified substrate; scale bar: 200 nm; (b) fluorescence images of the substrate immobilized with SH-Cu^2+^-DNAzyme-sgc8c-FAM and SH-Mg^2+^-DNAzyme-TD05-Cy5; (c) fluorescence images of the substrate immobilized with Cu^2+^-DNAzyme-sgc8c-FAM and Mg^2+^-DNAzyme-TD05-Cy5. Scale bars: 100 μm.

The capture platform was fabricated as shown in Fig. 1a. The AuNPs with a diameter of 13 nm were used as the supporting substrate because it can provide anchoring sites for the capture element through Au–S chemistry and has good biocompatibility for cells. The 13 nm AuNPs were fixed on the amino group modified glass surface *via* Au–N interaction and the modification was characterized by scanning electron microscopy (SEM). As shown in Fig. 2a, the 13 nm AuNPs were distributed uniformly and densely on the substrate. After the decoration of AuNPs on the substrate, the capture elements of Cu^2+^-DNAzyme-sgc8c and Mg^2+^-DNAzyme-TD05 were immobilized on the substrate through Au–S bonds. We modified the substrate strands of Cu^2+^-DNAzyme-sgc8c with FAM at the 5′ end and the substrate strands of Mg^2+^-DNAzyme-TD05 with Cy5 at the 3′ end. It can be seen that after immobilization of the thiol-modified capture elements, FAM and Cy5 fluorescence were emitted on the substrate (Fig. 2b) and when the capture elements without an –SH group were used, there was negligible fluorescence on the substrate (Fig. 2c), which suggested the successful modification of the capture elements. In order to prevent nonspecific adsorption, the substrate was further blocked with SH-PEG.^44,67^

### Cleavage verification by metal ions on the substrate

The selective release of multiple CTCs was realized *via* the catalytic cleavage of DNAzymes with metal ions as cofactors in our study. To verify the feasibility, gel electrophoresis was conducted to characterize this mechanism (Fig. S1†). We adopted 4 μM Cu^2+^ and 10 mM Mg^2+^ to catalyze the cleavage of the capture elements according to previous reports.^68,69^ As shown in Lane 2, the product of 4 μM Cu^2+^ catalyzed Cu^2+^-DNAzyme-sgc8c probes migrated faster than Cu^2+^-DNAzyme-sgc8c alone (Lane 1), indicating the successful cleavage of DNAzyme probes. Similarly, the product of 10 mM Mg^2+^ catalyzed Mg^2+^-DNAzyme-TD05 probes (Lane 4) migrated faster than Mg^2+^-DNAzyme-TD05 alone (Lane 3). The result demonstrates that Cu^2+^ and Mg^2+^ can be potent tools for the release of CTCs.

### Cell capture study

**Fig. 3:**
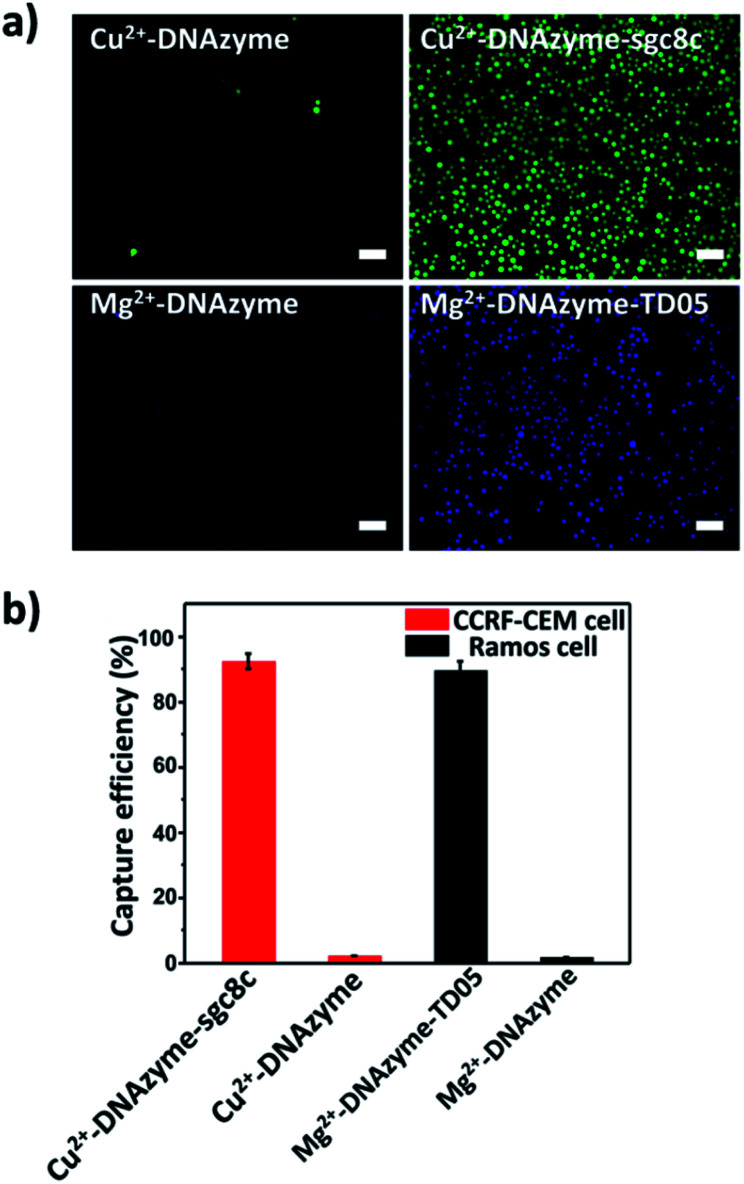
(a) Fluorescence images: CTC capture capability study of our proposed platform using different capture elements on the substrate; scale bars: 50 μm; (b) capture efficiencies of CTCs by different capture elements.

To explore the capture performance of our platform, we used CCRF-CEM cells and Ramos cells as the target cell lines. First, we studied the single cell type capture ability of our proposed capture platform. We prepared the Cu^2+^-DNAzyme-sgc8c modified substrate and the Mg^2+^-DNAzyme-TD05 modified substrate respectively. The Cu^2+^-DNAzyme-sgc8c modified substrate was incubated with CCRF-CEM cells and the Mg^2+^-DNAzyme-TD05 modified substrate was incubated with Ramos cells. In order to observe the cells clearly, CCRF-CEM cells were stained with calcein AM for 10 min while Ramos cells were stained with DAPI 15 min after incubation. As shown in Fig. 3a, the substrate without sgc8c aptamer or TD05 aptamer modification showed negligible capture of the corresponding CTCs compared to that of aptamer-containing element modified substrates. The large view of capture is shown in Fig. S2.† To optimize the capture ability of the platform, we investigated the capture time and concentration of the capture elements. As illustrated in Fig. S3,† with the increase of incubation time, the number of CTCs captured by the two capture elements all increased and reached saturation at 30 min. When the concentration of the two capture elements increased, the capture efficiency of Cu^2+^-DNAzyme-sgc8c for CCRF-CEM cells and Mg^2+^-DNAzyme-TD05 for Ramos cells all increased from 0.1 to 1 μM and reached a maximum at 1 μM (Fig. S4†). The capture efficiency of Cu^2+^-DNAzyme-sgc8c for CCRF-CEM cells was calculated to be 92 ± 2.4% within 30 min and the capture efficiency of Mg^2+^-DNAzyme-TD05 for Ramos cells was calculated to be 89 ± 2.8% within 30 min (Fig. 3b). In addition, the selectivity of the capture elements for CTCs was investigated. As shown in Fig. S5a,† the number of Ramos cells captured by Cu^2+^-DNAzyme-sgc8c is much less than that captured by Mg^2+^-DNAzyme-TD05. Meanwhile, the number of CCRF-CEM cells captured by Mg^2+^-DNAzyme-TD05 is much less than that captured by Cu^2+^-DNAzyme-sgc8c. Moreover, we chose K562 cells which have little binding affinity to the sgc8c aptamer and TD05 aptamer to further confirm the specificity of the two capture elements. As shown in Fig. S5b,† Cu^2+^-DNAzyme-sgc8c and Mg^2+^-DNAzyme-TD05 had almost no capture capability to K562 cells. Based on the above results, the binding capability between Cu^2+^-DNAzyme-sgc8c and CCRF-CEM cells and Mg^2+^-DNAzyme-TD05 and Ramos cells was demonstrated to be specific.

### Cell release study

**Fig. 4:**
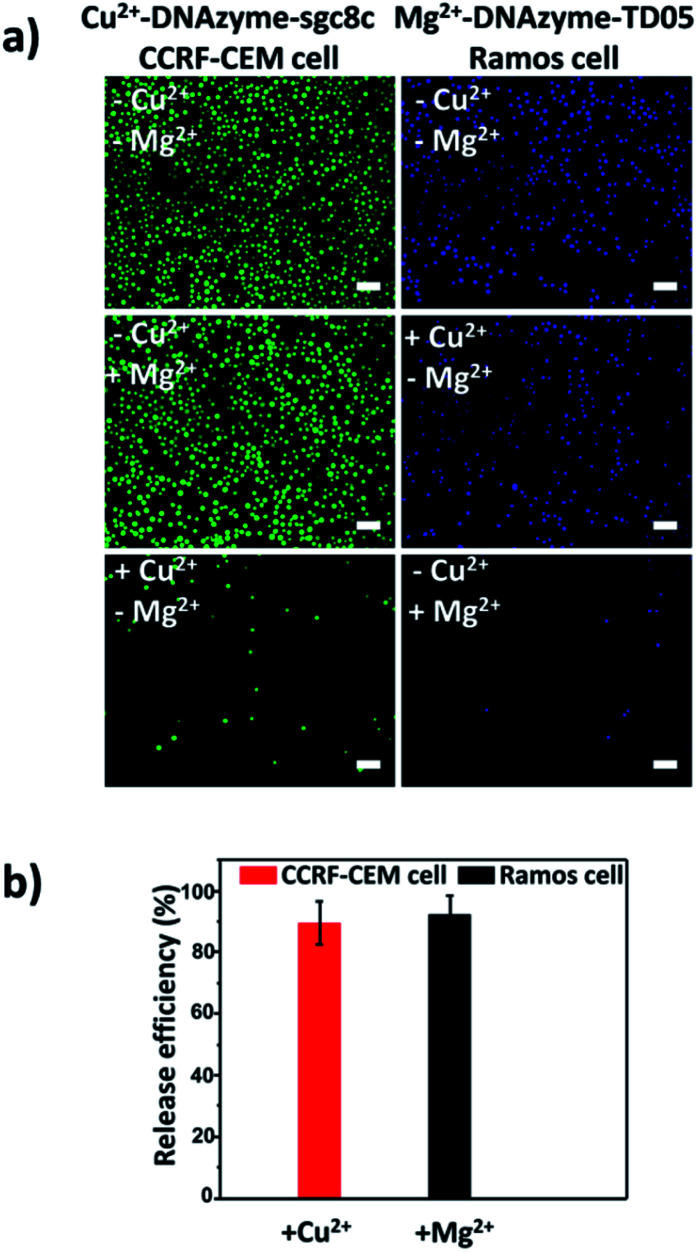
CTC release from the substrate triggered by metal ions. (a) Fluorescence images of CCRF-CEM cells and Ramos cells released from the substrate stimulated using different metal ions; scale bars: 50 μm; (b) release efficiencies of Cu^2+^-induced CCRF-CEM cells and Mg^2+^-induced Ramos cells.

Having successfully demonstrated the specific capture, we further testified the cell release capability of our proposed platform. After the capture of CCRF-CEM cells by the Cu^2+^-DNAzyme-sgc8c modified substrate, Cu^2+^ was added and incubated for 30 min to release CCRF-CEM cells. It can be observed that most CCRF-CEM cells were released from the substrate (Fig. 4a). In contrast, without the addition of Cu^2+^ or with the addition of Mg^2+^, only a negligible amount of CCRF-CEM cells were released from the substrate (Fig. 4a). Similarly, after the capture of Ramos cells by the Mg^2+^-DNAzyme-TD05 modified substrate, Mg^2+^ was added for 30 min to release Ramos cells. It can be concluded that most Ramos cells were released from the substrate only after the addition of Mg^2+^ (Fig. 4a). In contrast, the control samples, either without the addition of Mg^2+^ or with the addition of Cu^2+^, showed negligible Ramos cell release from the substrate (Fig. 4a). The large view of cell release is shown in Fig. S6.† Therefore, Cu^2+^ and Mg^2+^ as triggers can release CCRF-CEM cells and Ramos cells selectively. In order to obtain more effective release, we optimized the incubation time for the two metal ions to release the two CTCs.

As shown in Fig. S7,† with the increase of the incubation time of Cu^2+^, the release efficiency of CCRF-CEM cells increased and reached the maximum (89 ± 6.8%) (Fig. 4b) at 30 min and with the increase of the incubation time of Mg^2+^, the release efficiency of Ramos cells increased and reached the maximum (92 ± 6.2%) (Fig. 4b) at 50 min. Consequently, 30 min was chosen for Cu^2+^ to release CCRF-CEM cells, while 50 min was chosen for Mg^2+^ to release Ramos cells.

### Capture and selective release of multiple CTCs

**Fig. 5:**
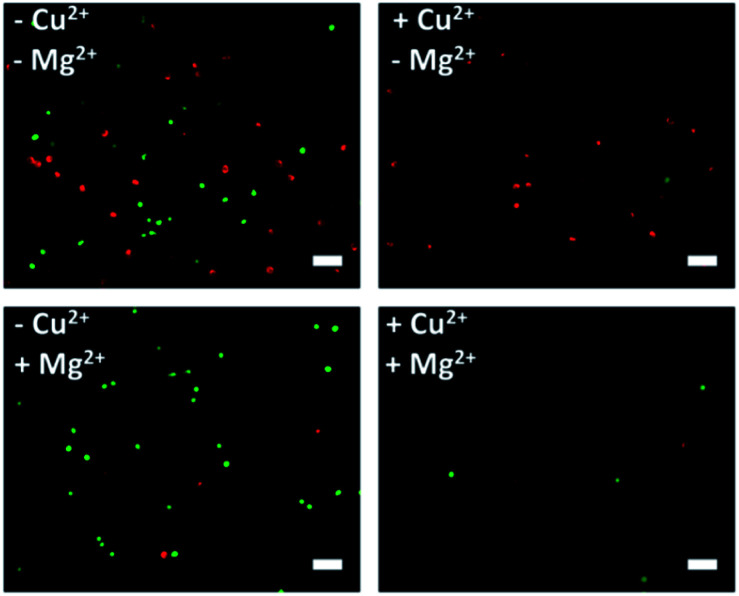
Fluorescence images of multiple CTCs after capture and selective release. The CCRF-CEM cells pre-stained with calcein AM (green) and Ramos cells pre-stained with DiI (red). Scale bars: 50 μm.

After corroborating the ability of our proposed platform for the capture and selective release of the two CTCs respectively, we modified Cu^2+^-DNAzyme-sgc8c and Mg^2+^-DNAzyme-TD05 on the same substrate to study simultaneous capture and selective release of multiple CTCs. We employed the optimal capture and release conditions that are illustrated in Fig. S3, S4 and S7.† CCRF-CEM cells were pre-dyed with calcein AM for 10 min and Ramos cells were pre-dyed with DiI for 30 min. The whole process of capture and release took time that DiI was chosen which had less cytotoxicity than DAPI. As shown in Fig. 5, CCRF-CEM cells and Ramos cells can be captured on the substrate efficiently before the addition of metal ions. When only Cu^2+^ was added, the majority of CCRF-CEM cells were released, while Ramos cells were rarely released. Similarly, when only Mg^2+^ was added, nearly all Ramos cells were released completely, leaving most of the CCRF-CEM cells on the substrate. In the presence of Cu^2+^ and Mg^2+^, nearly all of CCRF-CEM cells and Ramos cells were released from the substrate. The capture and selective release of two types of CTCs in large view are shown in Fig. S8.† Based on the above results, we can conclude that our design not only achieves the efficient capture of multiple CTCs, but also selective releases the CTCs, which was worthwhile for the subsequent study.

### Cytotoxicity of metal ions and the cell viability after release

To study the influence of metal ions on the cell viability, MTT assay was conducted. According to Fig. S9a and b,† it is clear that the viability of both CCRF-CEM cells and Ramos cells was maintained under 4 μM Cu^2+^ and 10 mM Mg^2+^, which was used to release CTCs, indicating that the concentration of Cu^2+^ and Mg^2+^ used in the release experiment had good biocompatibility. After the successful release of CTCs by metal ions, we explored the viability of released CTCs with live/dead cell staining with calcein AM and propidium iodide (PI). As shown in Fig. S9c and d,† almost 80% of the released CCRF-CEM cells and 90% of the released Ramos cells all showed good viability, which was essential for subsequent research of CTCs.

### CTC capture and release in simulated blood samples

**Fig. 6:**
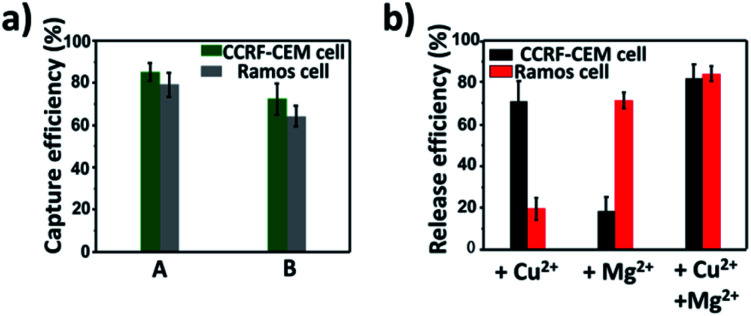
Capture and selective release of CTCs in the blood sample. (a) Capture efficiencies of CTCs in the blood sample spiked with different numbers of CTCs. A: 10^3^ CCRF-CEM cells per mL + 10^3^ Ramos cells per mL in blood; B: 10^2^ CCRF-CEM cells per mL + 10^2^ Ramos cells per mL in blood; (b) release efficiencies of different metal ion-induced CTCs' selective release from the substrate in the blood sample.

On the basis of the above results, calcein AM-stained CCRF-CEM cells and DiI-stained Ramos cells were mixed with a buffy coat layer of healthy human blood sample at a concentration of 10^3^ cells per mL to 10^2^ cells per mL to investigate the practicality of our strategy in mimic clinical samples. We used a buffy coat layer of healthy human blood sample because CTCs were in the buffy coat layer and the interference of erythrocyte and most non-specific proteins in the blood sample could be avoided.^70–72^ As shown in Fig. 6a, when 10^3^ CCRF-CEM cells and 10^3^ Ramos cells were spiked into 1 mL healthy human blood, the capture efficiency using our platform was 85 ± 4.1% for CCRF-CEM cells and 79 ± 5.9% for Ramos cells respectively. In the case of 10^2^ CCRF-CEM cells and 10^2^ Ramos cells in 1 mL healthy human blood, 72 ± 7.5% of CCRF-CEM cells were captured and 64 ± 4.8% of Ramos cells were captured. The capture efficiency of CTCs slightly decreased as the number of CTCs in the blood reduced, which was consistent with previous reports.^44,73^ Besides, by means of the modification of SH-PEG, the adhesion of the blood cells on the substrate was little during the capture of CTCs (Fig. S10†). To further confirm that the captured cancer cells were CTCs, we subjected the captured CCRF-CEM cells (pre-stained with DiI) and Ramos cells (pre-stained with Calcein AM) to immunofluorescence staining with FITC-labelled anti-CD3 (a marker for T cells) and APC-labelled anti-CD19 (a marker for B cells).^24^ As shown in Fig. S11,† DiI-stained CCRF-CEM cells showed positive for CD3 and calcein AM-stained Ramos cells showed positive for CD19, which suggested that the captured cancer cells were CTCs. These results clearly demonstrate that our capture platform can capture CTCs efficiently even in a whole blood sample, which is a relatively complex system.

Furthermore, we testified the selective release of CTCs through the addition of metal ions followed by the capture of two CTCs in a blood sample. As depicted in Fig. 6b, only in the presence of Cu^2+^, the release efficiency of CCRF-CEM cells was 71 ± 9.9% and the release efficiency of Ramos cells was 19 ± 5.3%. When only Mg^2+^ was added, the release efficiency of Ramos cells was 72 ± 3.9% and the release efficiency of CCRF-CEM cells was 18 ± 7.2%. The release of Ramos cells with the addition of Cu^2+^ and CCRF-CEM cells with the addition of Mg^2+^ came from the inevitable non-specific release caused by D-PBS flushing. In the presence of Cu^2+^ and Mg^2+^, the release efficiency was 82 ± 6.5% for CCRF-CEM cells and 85 ± 3.7% for Ramos cells respectively. These results confirmed that our work can not only provide a method to capture multiple CTCs adequately even in the blood samples but also to release CTCs selectively with high efficiency for further study.

### CTC isolation from cancer patients' blood samples

**Fig. 7:**
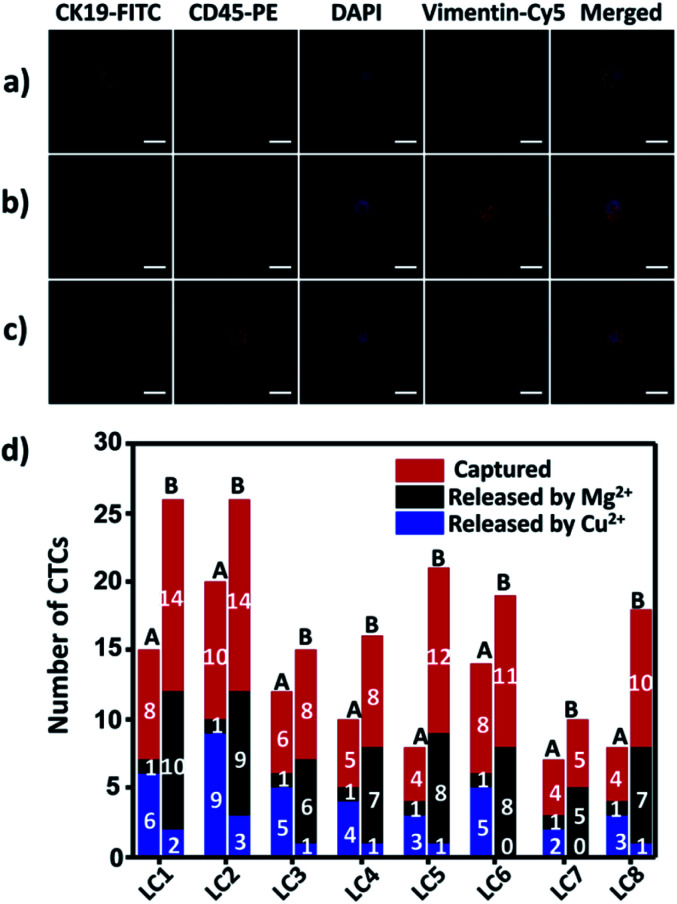
Isolation of CTCs from lung cancer (LC) patient blood samples. (a and b) Confocal images of CTCs captured from lung cancer patient blood samples with immunofluorescence staining; (c) confocal images of WBCs non-specifically captured from lung cancer patient blood samples with immunofluorescence staining; (d) captured and selectively released CTC counts from 0.5 mL of lung cancer patient blood samples. A: vimentin-positive CTCs; B: CK19-positive CTCs. Scale bars: 10 μm.

To demonstrate the suitability of our method for real clinical samples, we replaced the Sgc8c aptamer and the TD05 aptamer with the Lc-17 aptamer and the AP-1 aptamer to target two CTCs with abnormal expression of vimentin and with abnormal expression of cytokeratin (CK) respectively in lung cancer patients.^38,74–76^ After CTCs were captured by our platform, Cu^2+^ and Mg^2+^ were added to release CTCs successively. The released CTCs by Cu^2+^ or Mg^2+^ and unreleased CTCs were collected separately for immunofluorescence staining. As shown in Fig. 7a–c, there were vimentin-positive CTCs and CK19-positive CTCs and CD45-positive WBCs which were non-specifically captured simultaneously in a lung cancer patient blood sample. We calculated CTCs that were only positive for vimentin and CTCs that were only positive for CK19. The results summarized from 8 lung cancer patient blood samples are provided in Fig. 7d, which indicated that our strategy could perform well in real clinical samples.

## Experimental

### Materials

All the oligonucleotides were provided by Sangon Biotechnology Co. Ltd. (Shanghai, China), and all the sequences are shown in Table S2.† (3-aminopropyl)triethoxysilane (APTES) and tris(2-carboxyethyl)phosphine hydrochloride (TCEP) were purchased from Aladdin Reagent Co., Ltd. (China). BSA was obtained from Sigma-Aldrich (USA). 3-(4,5-dimethyl-2-thiazolyl)-2,5-diphenyl-2*H*-tetrazolium bromide (MTT), DAPI and DiI were purchased from KeyGEN BioTECH Co., Ltd. (Nanjing, China). Calcein AM and propidium iodide (PI) were bought from Dojindo Molecular Technologies. SH-PEG (molecular weight: 1000) was provided by Yare Biotech, Co., Ltd. (Shanghai, China). APC-labeled mouse anti-human CD19 (CD19-APC), FITC-labeled mouse anti-human CD3 (CD3-FITC), PE-labeled mouse anti-human CD45 (CD45-PE), mouse anti-cytokeratin 19 antibody (CK19), FITC-labeled goat anti-mouse lgG antibody, rabbit anti-vimentin antibody and Cy5-labeled goat anti-rabbit lgG antibody were purchased from Bioss (Beijing, China). Ultrapure water was used in all experiments. All chemicals were of analytical grade and used without any further purification. All experiments using human samples were performed in accordance with the Guidelines of “the 1975 Declaration of Helsinki Principles” and experiments were approved by the Ethical Committee of Medical Research, Nanjing Drum Tower Hospital, Affiliated Hospital of Nanjing University Medical School. Informed consents were obtained from human participants of this study.

### Instrumentation

Field-emission scanning electron microscopy (FE-SEM) images were obtained on a Hitachi S-4800 scanning electron microscope (Hitachi Co., Japan). The fluorescence images were recorded on a fluorescence inversion microscope system (Nikon, TI-U). Confocal images were obtained from confocal laser scanning microscopy (CLSM) on a Leica TCS SP8 confocal microscope (Leica Microsystems Inc., Exton, PA). The cell number was counted with a Petroff-Hausser cell counter (USA). Cell viability assay was performed on a Varioskan Flash microplate reader (ThermoFisher Scientific).

### Preparation of the substrate

4 mm × 4 mm glass slides were used as the substrate. First, the glass slides were immersed in piranha solution for 12 h and then rinsed with plenty of Milli-Q water. After drying with nitrogen, the glass slides were soaked in 0.5% (3-aminopropyl)triethoxysilane (APTES) solution for 10 h and rinsed with ethanol and Milli-Q water successively. Finally, the glass slides were grilled in a vacuum drying oven for 2 h.

### Fabrication of the capture platform

AuNPs with a diameter of 13 nm were prepared according to a reported method previously.^77^ The aminated substrate was incubated with the solution of 13 nm AuNPs for 2 hour at room temperature. Then the resulting substrate was washed with Milli-Q water and D-PBS successively. The thiolated DNAzyme strand was activated with TCEP at a molar ratio of 1 : 100 for 1 hour at room temperature and then mixed with the substrate strand at a molar ratio of 1 : 1 in buffer A or buffer B. Buffer A (1.5 M NaCl, 50 mM HEPES, pH 7.0) was used for Cu^2+^-DNAzyme-sgc8c and buffer B (150 mM NaCl, 50 mM Tris–HCl, pH 7.5) was used for Mg^2+^-DNAzyme-TD05. After annealing, 80 μL of different concentrations of the mixture of the DNAzyme strand and substrate strand was incubated with the AuNP modified substrate for 2 hour at room temperature. After washing with D-PBS, the obtained substrate was incubated with 200 μL of 5 μM SH-PEG (molecular weight: 1000) for 1 hour at room temperature to block the residual nonspecific binding sites. After incubation for 1 hour, the obtained substrate was rinsed with D-PBS and employed as the capture platform. The capture elements modified with FAM or Cy5 should be protected from light during the modification process.

### Polyacrylamide gel electrophoresis

The metal ions could cleave the corresponding capture DNA, which was verified using 12% polyacrylamide gel. 10 μL of 1 μM sample was loaded onto 12% polyacrylamide gel for electrophoresis at 150 V for 45 min in 1× TBE buffer. The gel was stained with ethidium bromide and imaged with a fluorescence gel imaging system.

### Cell culture

Ramos cells, CCRF-CEM cells and K-562 cells were all obtained from KeyGen Biotech. Co. Ltd. (Nanjing, China). They were all cultured in Roswell Park Memorial Institute 1640 medium (KeyGen Biotech. Co. Ltd Nanjing, China) supplemented with 10% fetal calf serum (Gibco, Grand Island, NY), penicillin (100 μg mL^−1^) and streptomycin (100 μg mL^−1^) in an incubator (5% CO_2_, 37 °C).

### Cell capture

Before experiments, cells were distributed in the binding buffer (1 mg mL^−1^ BSA, 4.5 g L^−1^ glucose in D-PBS) and diluted to a concentration of 1 × 10^6^ cells per mL. Then, cell suspensions (200 μL, 1 × 10^6^ cells per mL) were incubated with the capture platform in an incubator (5% CO_2_, 37 °C) for different times. The surface of the substrate was rinsed with D-PBS gently to remove the unbound cells. CCRF-CEM cells were stained with calcein AM for 10 min while Ramos cells were stained with DAPI 15 min in an incubator (5% CO_2_, 37 °C). The capture results of CTCs were viewed using an inverted fluorescence microscope system. For the capture of CCRF-CEM cells and Ramos cells simultaneously, 200 μL of 1 × 10^6^ cells per mL CCRF-CEM cells pre-dyed with calcein AM and Ramos cells pre-dyed with DiI were incubated with the capture platform in an incubator (5% CO_2_, 37 °C) for 30 min.

### Selective cell release by metal ions

To release the CCRF-CEM cells or Ramos cells, 4 μM Cu^2+^ and 50 μM ascorbate or 10 mM Mg^2+^ was added to the corresponding capture platform for different times in an incubator (5% CO_2_, 37 °C). After release, the substrate was gently washed with D-PBS for 20 s to remove the released cells and the metal ions. For the release of the two CTCs respectively, CCRF-CEM cells were stained with calcein AM for 10 min while Ramos cells were stained with DAPI 15 min finally. For the selective release of CCRF-CEM cells and Ramos cells simultaneously, CCRF-CEM cells were pre-dyed with calcein AM for 10 min and Ramos cells were pre-dyed with DiI for 30 min. The release results of CTCs were viewed using an inverted fluorescence microscope system.

### Statistics of capture efficiency and release efficiency

For calculating the capture efficiency and release efficiency, the 4 mm × 4 mm substrate was controlled using a PDMS film to complete capture and release according to the abovementioned method. The numbers of cells captured and released was counted using an inverted fluorescence microscope system.

### Cytotoxicity of metal ions and the cell viability after release

The cytotoxicity of metal ions was assessed using a 3-(4,5)-dimethylthiahiazo(-z-y1)-3,5-diphenytetrazoliumromide (MTT) assay using CCRF-CEM cells and Ramos cells. In short, the CCRF-CEM cells or Ramos cells (100 μL, 1 × 10^5^ cells per mL) were seeded into a 96-well cell-culture plate, followed by the incubation of metal ions at an appropriate concentration. Then the cells were cultured for 24 h in an incubator (5% CO_2_, 37 °C). After discarding the supernatant by centrifugation, 100 μL of MTT (0.5 mg mL^−1^) was added to each well and incubated for 4 h. After 4 h, the supernatant was removed by centrifugation, and 100 μL DMSO was added to each well. Then the optical density (OD) was read at a wavelength of 490 nm. Relative cell viability was expressed as follows: % = ([OD]_test_/[OD]_control_) × 100. The viability of the released cells was assayed with live/dead cell staining with calcein AM and propidium iodide (PI). 2 μM calcein AM and 4 μM PI were incubated with the released cells for 15 min in an incubator (5% CO_2_, 37 °C). And then the cells were observed using an inverted fluorescence microscope system.

### CTC capture and release in blood samples

The buffy coat layer of the blood sample was collected carefully. After centrifugation at 450*g* for 10 min, the supernatant was discarded. The sediment was resuspended in 10 volumes of ACK lysis buffer (150 mM NH_4_Cl, 10 mM KHCO_3_, 0.1 mM EDTA) and incubated at 4 °C for 10 min to remove the erythrocytes. Then the mixture was centrifuged for another 10 minutes, and the cell pellets were collected carefully and resuspended in the original volume of the binding buffer. Mimic clinical samples were obtained by mixing CCRF-CEM cells pre-dyed with calcein AM and Ramos cells pre-dyed with DiI with pretreated blood sample at 10^3^ cells per mL or 10^2^ cells per mL. And the mimic clinical samples or cancer patient samples were incubated with the 4 mm *×* 4 mm capture platform controlled with a PDMS film for 30 min in an incubator (5% CO_2_, 37 °C). The capture results of CTCs were viewed using an inverted fluorescence microscope system. The release process followed the method mentioned earlier.

### Immunofluorescence staining

Cells collected in 96-well plates were fixed in 4% paraformaldehyde for 30 min at room temperature. After washing with PBS three times by centrifugation at 2300 rpm for 10 min, the cells were blocked in D-PBS containing 5% FCS for 30 min at room temperature and washed three times with D-PBS. Then the cells were incubated with the primary antibody overnight at 4 °C. For primary antibodies with the fluorophore conjugated, the cells were washed three times with D-PBS and viewed by confocal microscopy imaging. For primary antibodies without the fluorophore conjugated, the cells were incubated with secondary antibodies for 2 h at room temperature after washing with PBS three times. Then the cells were viewed by confocal microscopy imaging after washing with D-PBS three times.

## Conclusions

In summary, we have reported a strategy to realize the capture and selective release of multiple CTCs using smart metal ion responsive DNAzymes probes. Our capture platform can capture multiple CTCs simultaneously with approximately 90% efficiency in buffer and with 80% efficiency in the blood sample. In addition, compared with the methods reported to capture multiple CTCs, we utilized metal ions to release CTCs that are not only more economical. Meanwhile it can achieve selective release of multiple CTCs captured with the potential to obtain downstream molecular information of a specific CTC. It has great potential to promote the development of individualized treatment of cancer.

## Conflicts of interest

There are no conflicts to declare.

## Supplementary Material

SC-011-C9SC04309H-s001
