# Supplementary material for: Capture and selective release of multiple types of circulating tumor cells using smart DNAzyme probes
Source: Chem Sci. 2020 Jan 9;11(7):1948–56. doi: 10.1039/c9sc04309h (PMC8148068; doi:10.1039/c9sc04309h)
Supplement: SC-011-C9SC04309H-s001 [file SC-011-C9SC04309H-s001.pdf]

Electronic Supplementary Material (ESI) for Chemical Science.
This journal is © The Royal Society of Chemistry 2020

Supporting information

Capture and Selective Release of Multiple Types of Circulating Tumor Cells Using Smart DNAzyme Probes

Qianying Zhang,‡[a] Wenjing Wang,‡[c] Shan Huang,[a] Sha Yu,[a] Tingting Tan,[d] Jian-Rong Zhang,*[ab] and Jun-Jie Zhu*[a]

[a] State Key Laboratory of Analytical Chemistry for Life Science, School of Chemistry and Chemical Engineering, Nanjing University, Nanjing 210023, China.

[b] School of Chemistry and Life Science, Nanjing University Jinling College, Nanjing 210089, China.

[c] State Key Laboratory of Agricultural Microbiology, College of Science, Huazhong Agricultural University, Wuhan 430070, China.

[d] Department of Laboratory Medicine, Nanjing Drum Tower Hospital, The Affiliated Hospital of Nanjing University Medical School, Nanjing, 210008, China.

* Corresponding Authors: jrzhang@nju.edu.cn; jjzhu@nju.edu.cn.

‡ Q. Z. and W. W. contributed equally to this work.

Supporting Tables

Table S1 Comparison with CTC separation methods reported

Approach	Targeted marker	Multiple CTCs	Capture efficiency	Patient blood sample	Release methods	Selective release
**Immunomagnetic separation**[1]	Anti-EpCAM	No	90%	Yes	Photocontrolled release	No
**Immunomagnetic separation**[2]	Anti-EpCAM	No	96%	Yes	No	No
**Microfluidic chip**[3]	Anti-EpCAM	No	>90%	Yes	Thiolated ligand-exchange	No
**Microfluidic chip**[4]	Anti-EpCAM	No	>95%	Yes	Temperature-responsive release and mechanosensitive release	Yes
**Magnetic separation**[5]	Sgc8c aptamer	No	88%	Mimic blood sample	DNase release	No
**Barcode-particle technology**[6]	Sgc8c, TD05 aptamers	Yes	>95%	Mimic blood sample	Exonuclease release	No
**Porous membranes separation**[7]	S6, A9 and YJ-1 aptamers	Yes	>95%	Mimic blood sample	Size-controlled release	No
**This work**	Sgc8c, TD05 aptamers	Yes	90%	Yes	Metal ions release	Yes

Table S2 Sequences of oligonucleotides used in the study

Name	Sequence(5'-3')
Cu^{2+}-DNAzyme	SH-$(CH_2)_6$-GGTAAGCCTGGGCCTCTTTCTTTTTAAGAAAGAAC
Cu^{2+}-substrate	AGCTTCTTTCTAATACGGCTTACC
Cu^{2+}-substrate-sgc8c	ATCTAACTGCTGCGCCGCCGGGAAAATACTGTACGGTTAGATTTTTTTTTTTTAGCTTCTTT CTAATACGGCTTACC
Mg^{2+}-DNAzyme	CCG CGG CCA GGC TAG CTA CAA CGA CCT GGA CGA TTTTTT-$(CH_2)_6$-SH
Mg^{2+}-substrate	TCGTCCAGGrArUGGCCGCGG
Mg^{2+}-substrate-TD05	TCGTCCAGGrArUGGCCGCGGTTTTTTTTTTTTAACACCGGGAGGATAGTTCGGTGGCTGT TCAGGGTCTCCTCCCGGTG
AP-1	TTTATGGGTGGGTGGGGGGTTTTT
LC-17	CTC CTC TGAC TGT AAC CACG CTT TTG TCTT TAG CCG AATT TTA CTA AGCC GGG CTG ATCA GCA TAG GTAG TCC AGA AGCC

Supporting Figures

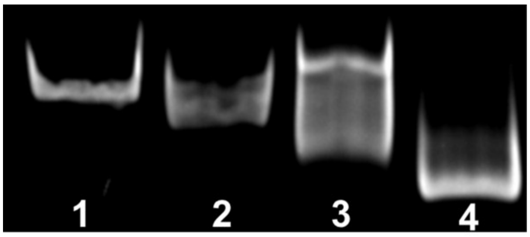

Figure. S1. EB-stained 12% polyacrylamide gel electrophoresis (PAGE) analysis of Cu^{2+}-DNAzyme-sgc8c and Mg^{2+}-DNAzyme-TD05 catalytic capabilities by metal ions. Lane 1: Cu^{2+}-DNAzyme-sgc8c strand; Lane 2: lane 1 + Cu^{2+}; Lane 3: Mg^{2+}-DNAzyme-TD05 strand; Lane 4: lane 3 + Mg^{2+}.

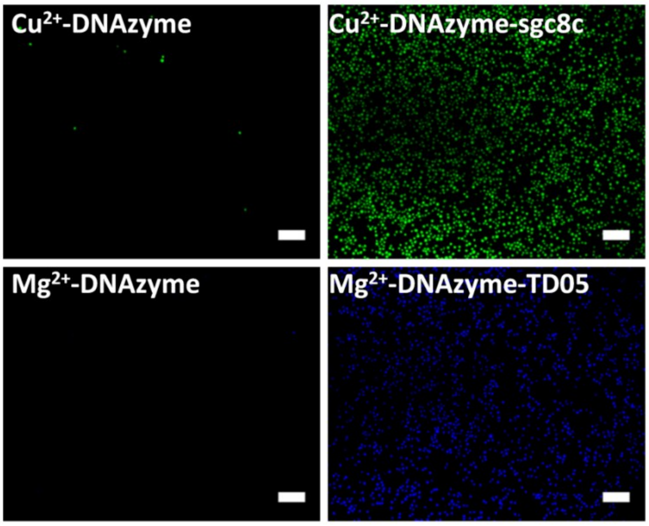

Figure. S2. Fluorescence images of CTCs captured by different capture elements on the substrate. Scale bars: 100 μm.

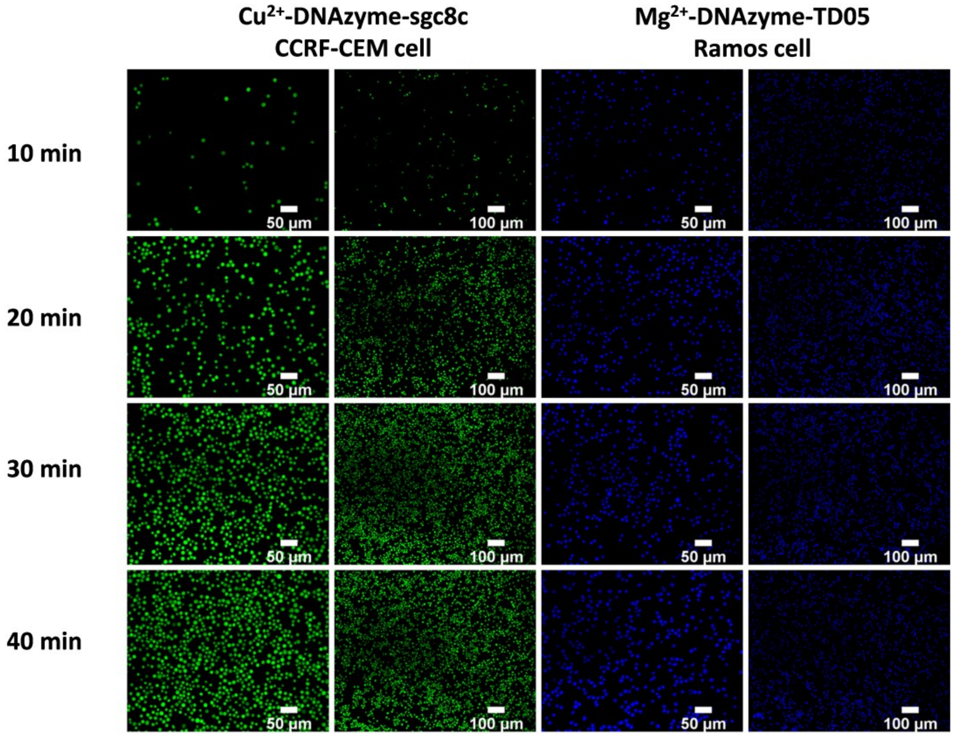

Figure. S3. Fluorescence images of CCRF-CEM cells captured by Cu^{2+}-DNAzyme-sgc8c and Ramos cells captured by Mg^{2+}-DNAzyme-TD05 with different incubation time.

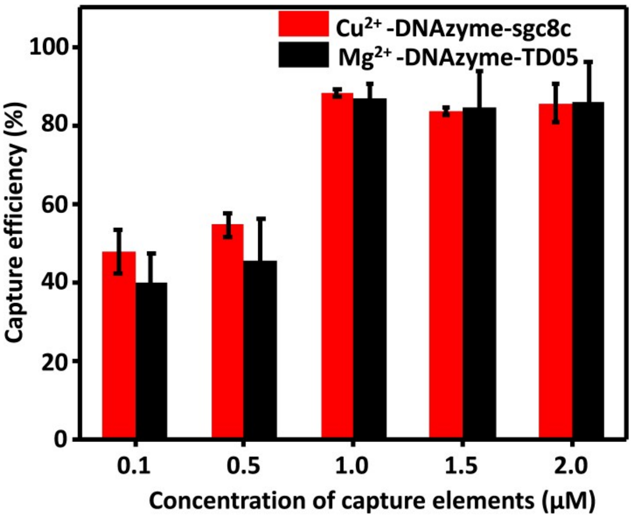

Figure. S4. Optimization of capture elements concentration used to capture CTCs.

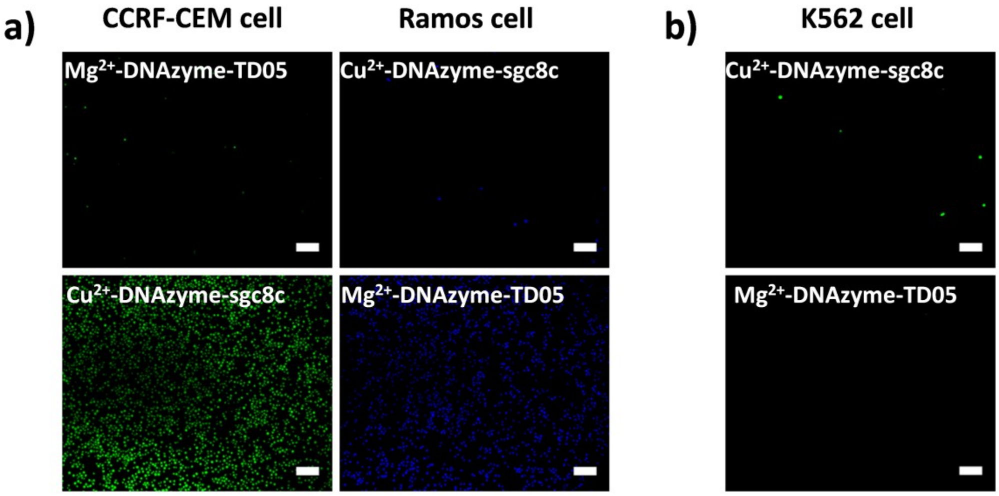

Figure. S5. Selectivity study of Cu^{2+}-DNAzyme-sgc8c and Mg^{2+}-DNAzyme-TD05 for CTCs. Scale bars: 100 µm.

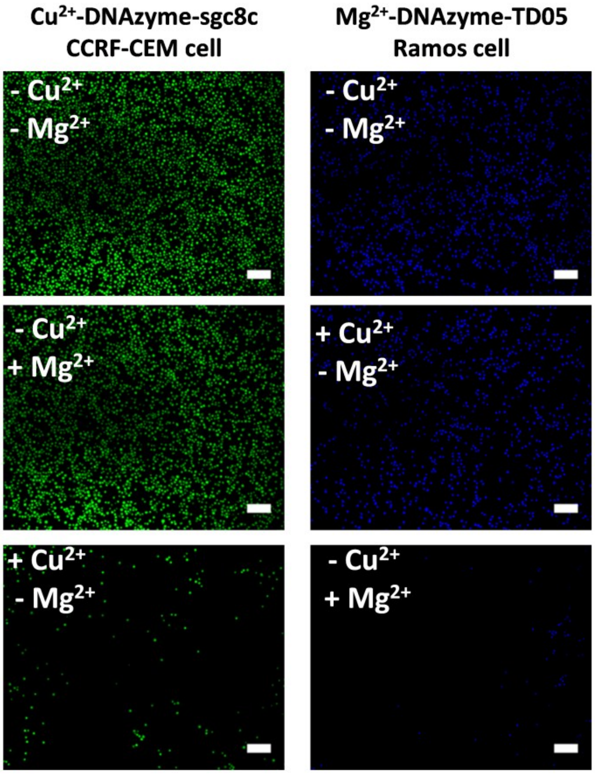

Figure. S6. Fluorescence images of CCRF-CEM cells and Ramos cells released from the substrate stimulated by different metal ions. Scale bars: 100 µm.

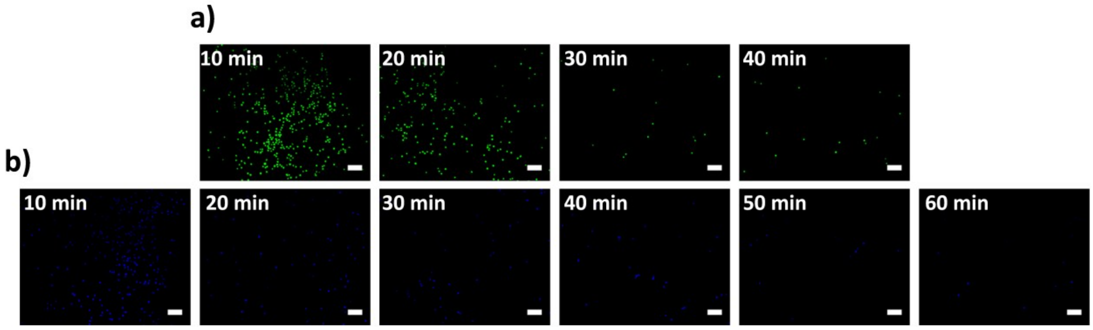

Figure. S7. Fluorescence images of CCRF-CEM cells release by Cu^{2+} and Ramos cells release by Mg^{2+} with different incubation time. Scale bars: 100 μm.

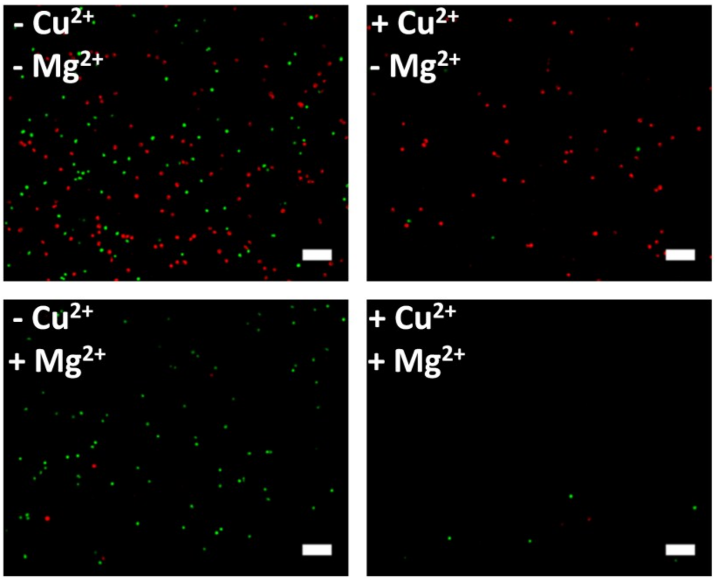

Figure. S8. Fluorescence images of multiple CTCs after capture and selective release. The CCRF-CEM cells pre-stained with Calcein AM (green) and Ramos cells pre-stained with DiI (red). Scale bars: 100 μm.

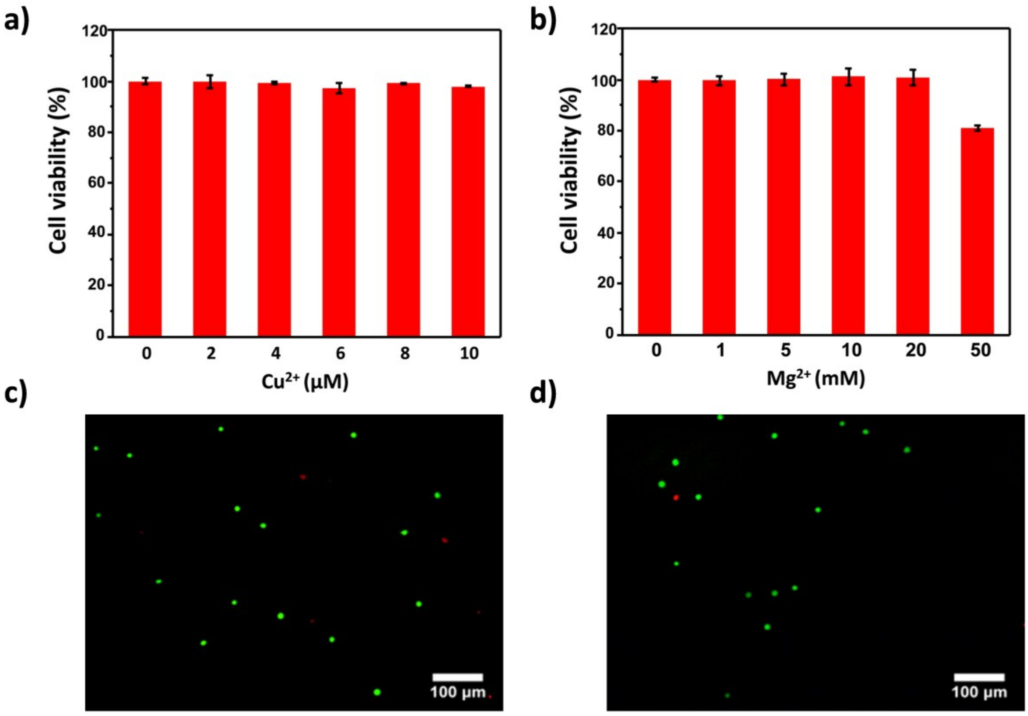

Figure. S9. (a) Cell viability of CCRF-CEM cells after incubation with different concentrations of Cu^{2+} for 24 h; (b) cell viability of Ramos cells after the treatment with different concentrations of Mg^{2+} for 24 h; (c and d) live (green)/dead (red) cell staining of the released CCRF-CEM cells by Cu^{2+} (c) and Ramos cells by Mg^{2+} (d).

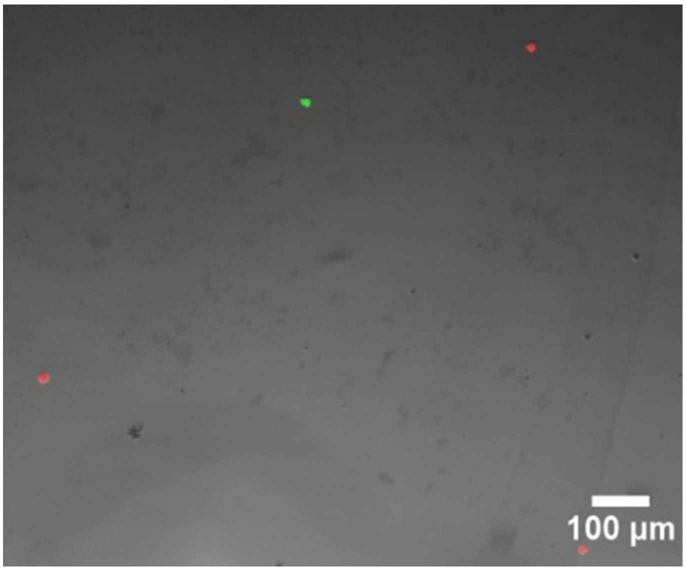

Figure. S10. Merged image of bright field and fluorescence of the captured CTCs on the substrate in blood sample.

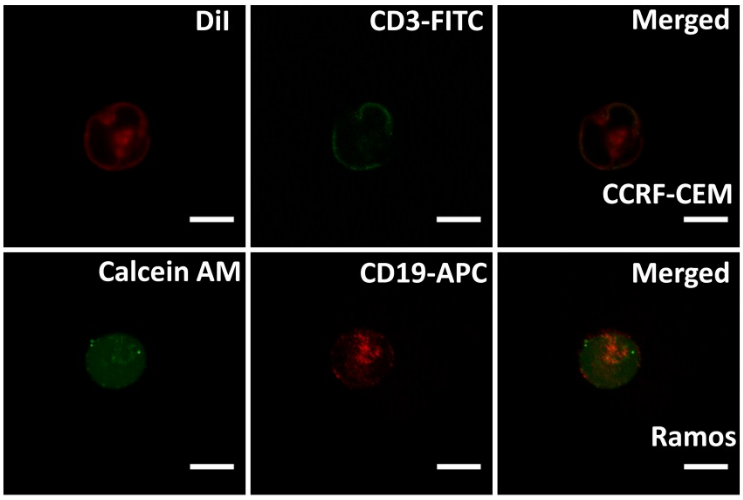

Figure. S11. Confocal images of CTCs captured from simulated blood sample with immunofluorescence staining. Scale bars: 10 μm.

References

1. S.-W. Lv, J. Wang, M. Xie, N.-N. Lu, Z. Li, X.-W. Yan, S.-L. Cai, P.-A. Zhang, W.-G. Dong and W.-H. Huang, *Chem. Sci.*, 2015, **6**, 6432-6438.
2. C.-Y. Wen, L.-L. Wu, Z.-L. Zhang, Y.-L. Liu, S.-Z. Wei, J. Hu, M. Tang, E.-Z. Sun, Y.-P. Gong, J. Yu and D.-W. Pang, *ACS Nano*, 2014, **8**, 941-949.
3. M.-H. Park, E. Reátegui, W. Li, S. N. Tessier, K. H. K. Wong, A. E. Jensen, V. Thapar, D. Ting, M. Toner, S. L. Stott and P. T. Hammond, *J. Am. Chem. Soc.*, 2017, **139**, 2741-2749.
4. E. Reátegui, N. Aceto, E. J. Lim, J. P. Sullivan, A. E. Jensen, M. Zeinali, J. M. Martel, A. J. Aranyosi, W. Li, S. Castleberry, A. Bardia, L. V. Sequist, D. A. Haber, S. Maheswaran, P. T. Hammond, M. Toner and S. L. Stott, *Adv. Mater.*, 2015, **27**, 1593-1599.
5. Z. Li, G. Wang, Y. Shen, N. Guo and N. Ma, *Adv. Funct. Mater.*, 2018, **28**, 1707152.
6. F. Zheng, Y. Cheng, J. Wang, J. Lu, B. Zhang, Y. Zhao and Z. Gu, *Adv. Mater.*, 2014, **26**, 7333-7338.
7. B. P. Viraka Nellore, R. Kanchanapally, A. Pramanik, S. S. Sinha, S. R. Chavva, A. Hamme, 2nd and P. C. Ray, *Bioconjugate Chem.*, 2015, **26**, 235-242.
